# Virtual Reality Body Exposure Therapy for Anorexia Nervosa. A Case Report With Follow-Up Results

**DOI:** 10.3389/fpsyg.2020.00956

**Published:** 2020-05-15

**Authors:** Bruno Porras-Garcia, Eduardo Serrano-Troncoso, Marta Carulla-Roig, Pau Soto-Usera, Marta Ferrer-Garcia, Natàlia Figueras-Puigderrajols, Lena Yilmaz, Yigit Onur Sen, Nazila Shojaeian, José Gutiérrez-Maldonado

**Affiliations:** ^1^Department of Clinical Psychology and Psychobiology, University of Barcelona, Barcelona, Spain; ^2^Department of Child and Adolescent Psychiatry and Psychology, Hospital Sant Joan de Déu Barcelona, Barcelona, Spain; ^3^Children and Adolescent Mental Health Research Group, Institut de Recerca Sant Joan de Déu, Barcelona, Spain

**Keywords:** anorexia nervosa, virtual reality, body-exposure therapy, fear of gaining weight, body image disturbance, case report

## Abstract

**Objective:**

Exposure-based therapies such as mirror exposure may help to improve the results of classic cognitive behavioral therapy in anorexia nervosa (AN). Virtual reality (VR)-based procedures provide interesting novelties for targeting body-related concerns. This study aimed to provide preliminary evidence of the usefulness of a VR body exposure therapy in a patient diagnosed with AN.

**Method:**

Fear of gaining weight (FGW), body anxiety, drive for thinness, body image disturbances, body mass index and body-related attentional bias were assessed before and after the intervention, as well as 5 months later. Five sessions of VR body exposure therapy were included within the standard course of cognitive behavioral therapy. The sessions involved a systematic and hierarchical exposure of the patient to a virtual representation of her own silhouette, with the body mass index of the avatar progressively increasing in subsequent sessions.

**Results:**

After the intervention, there was a clear reduction in AN symptoms such as the FGW, drive for thinness, body-related anxiety and dissatisfaction. Body mass index values rose continuously during the intervention and reached healthy levels. Finally, there was a notable change in the dysfunctional body-related attentional bias. Almost all these improvements were maintained after 5 months, except for the FGW.

**Conclusion:**

To the best of our knowledge, this study is the first to focus on treating the FGW and body-related concerns in AN using a VR-based paradigm. To pursue this study further and assess the effectiveness of this new VR software, larger controlled clinical trials are needed.

## Introduction

Anorexia nervosa (AN) affects approximately 1–4% of European women (Keski-Rahkonen and Mustelin, 2016) and is considered a serious mental health disorder. Adolescents and young adults are particularly at risk (Zipfel et al., 2015). Fear of gaining weight (FGW) and body image disturbances have been typically considered to be the core components of eating disorders (ED), including AN (DSM-5, American Psychiatric Association [APA], 2013).

Exposure-based therapies may help to improve the effects of classic cognitive behavioral therapy (CBT) for EDs (Murray et al., 2016; Reilly et al., 2017) by targeting body-related fears such as the FGW in AN patients (Reilly et al., 2017). Among *in vivo* exposure-based therapies, mirror exposure has been successfully used to reduce body dissatisfaction (BD) in ED patients (Hildebrandt et al., 2012), AN patients (Key et al., 2002), and individuals with high levels of BD (Jansen et al., 2016). To the best of our knowledge, only one case study has reported successful treatment of core FGW in a patient with restrictive AN using five sessions of imaginal exposure (Levinson et al., 2014). However, imaginal exposure may have some significant limitations, for example, difficulties in achieving or maintaining visualization and the risk that patients will deliberately avoid the most feared stimulus during visualization. These limitations can be overcome by the application of virtual reality (VR).

Virtual reality technologies may help improve the effects of body exposure therapy in ED. For example, they allow researchers to create real-size 3D simulations of the participants’ bodies by using their own physical characteristics and placing them in immersive VR environments that reproduce real-life situations related to their food- or body-related concerns (Pla-Sanjuanelo et al., 2017; Gutiérrez-Maldonado et al., 2018). In addition, the illusory feeling of ownership of the virtual body can be elicited by using embodiment-based procedures (e.g., Serino et al., 2016; Ferrer-Garcia et al., 2017; Porras Garcia et al., 2019). In this full-body illusion (FBI) participants feel the virtual body as their own. VR embodiment-based procedures have already been used in the assessment and treatment of body image disturbances in ED (for a more extensive review, see Gutiérrez-Maldonado et al., 2018). For instance, an interesting recent case report involving a patient with AN, showed that VR embodiment-based techniques (e.g., using a body swapping stimulation procedure) produced a short-term change in distorted body representations of specific body areas (Serino et al., 2019). The use of VR as an embodiment-based technology (Riva et al., 2019b) might not only improve dysfunctional body representations that individuals with ED have (Riva et al., 2019a), but also target core body-related fears in AN, such as the FGW.

To the best of our knowledge, this study is the first to focus on reducing the FGW and other body-related symptomatology in AN, using a VR-based paradigm. This study aimed to provide preliminary evidence of the usefulness of this innovative and unique body exposure technique in a patient with a DSM-5 diagnosis of AN.

A systematic and hierarchical exposure of the patient to a virtual representation of their own silhouette was performed, with the body mass index (BMI) of the avatar progressively increased in subsequent sessions. This was conducted over five sessions of VR body exposure therapy, which was part of the standard CBT given to the patient. Reductions in the FGW, body-related anxiety, body image disturbances and body-related attentional bias were expected, as well as a return to a healthy BMI. Finally, all these changes were expected to be maintained after 5 months.

## Case Formulation

Patient A is a 15-year-old woman diagnosed with restrictive AN according to the Diagnostic and Statistical Manual of Mental Disorders [DSM; 5th Ed., American Psychiatric Association [APA], 2013]. She was diagnosed at the Eating Disorders Unit of Hospital Sant Joan de Déu in Barcelona. The patient was referred to the Eating Disorders Unit by primary care providers after she had lost 10 kg in the previous 9 months and presented a BMI of 15.9 (percentile, 5%). In the previous year, with a maximum BMI value of 19.78 kg/m^2^, the patient started to present significant food restrictive behaviors, such as a progressively reduced intake or removal of some foods perceived to be unhealthy. These behaviors were accompanied by intense daily physical activity of around 2 or 3 h per day. However, she did not present typical purgative behaviors. Furthermore, she presented a high level of body image disturbances, showing concerns for certain weight-related body areas (e.g., the thighs) and a distorted body image.

The patient did not present any relevant personal or family history of psychopathology. She was in the third year of obligatory education, with very good grades, and lived with her family of origin. According to her medical records, her age at menarche was 12 years and she had amenorrhea 2 months before entering the Eating Disorders Unit. She had been previously diagnosed with severe iron deficiency anemia, for which she was prescribed a daily 40 mg dose of oral iron solution (iron proteinsuccinylate, FERPLEX). At the time of her admission into the Eating Disorders Unit, her hemoglobin levels had almost returned to normal (*Hb* = *12.8 g/dl*).

At the beginning of the treatment, she presented slight symptoms of asthenia, apathy and anhedonia, but did not meet the criteria for any other psychopathological diagnoses (e.g., a depressive episode). Although she was partly aware of her disorder, she was highly motivated to recover and complete the whole treatment.

**FIGURE 1:**
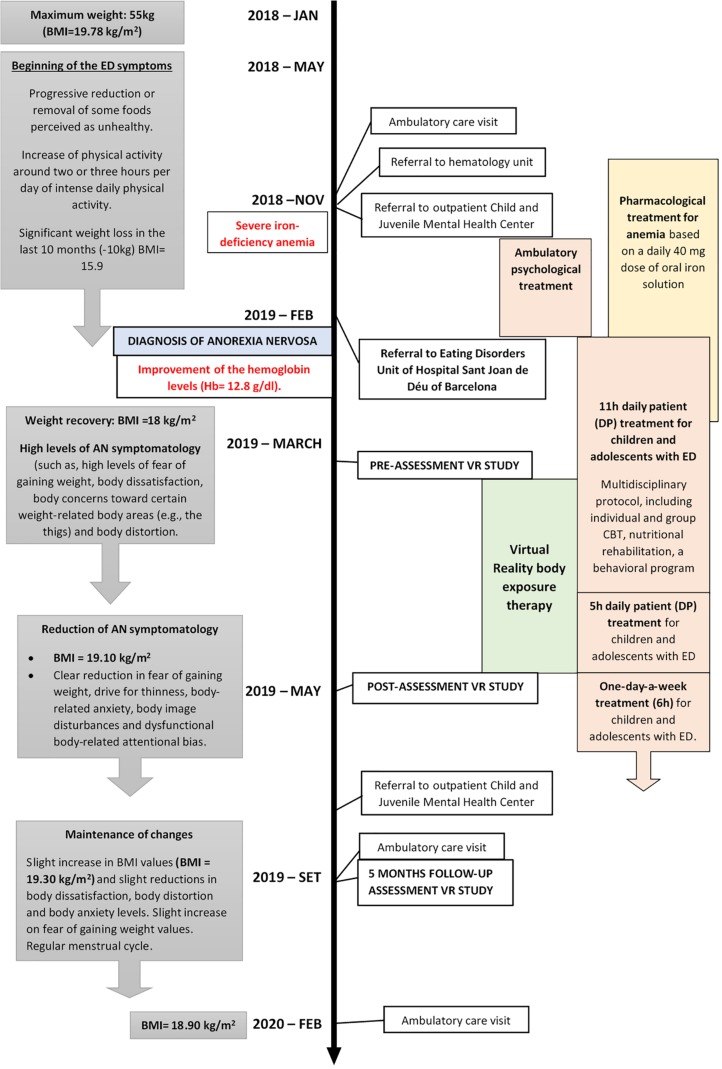
Relevant current and past clinical information of the patient organized into a timeline.

The patient underwent day patient (DP) treatment for children and adolescents with ED. It was an intensive treatment program conducted at the Eating Disorders Unit over 11-hour and 5-hour periods, with permission to sleep at home. For a more detailed description, please see Serrano-Troncoso et al. (2020). Treatment consisted of a multidisciplinary protocol, including individual and group CBT, nutritional rehabilitation, a behavioral program that aimed to improve eating patterns and increase weight, and individual and group (parent) counseling. The individual CBT consisted of two sessions per week, each lasting 45 min. The relevant current and past clinical information of the patient is shown as a timeline in Figure 1.

## Method

### Measures

All assessments were performed before starting the VR exposure treatment (pre-assessment), at the end of the treatment (post-assessment) and after 5 months.

#### AN Symptomatology

- BMI. Change in body weight was evaluated by weighing and measuring the patient and subsequently calculating her BMI.
- Eating Disorder Inventory (EDI-3; Garner, 2004). The EDI-3 is a self-report inventory consisting of 12 scales and 91 items, in which the answers are provided on a 6-point Likert scale. In the current study, only the 10-item BD subscale (EDI-BD) and 7-item Drive for Thinness subscale (ED-DT) were used. The Spanish version of the EDI-3 has robust validity indices and good internal consistency (Elosua et al., 2010).

#### Body Anxiety and Body Image Disturbances

- Physical Appearance State and Trait Anxiety Scale – PASTAS (Reed et al., 1991). The PASTAS comprises two self-report scales measuring weight-related and non-weight-related anxiety. In this study, the Weight Scale (W) was used. The questionnaire presents good reliability and convergent validity indices.
- Silhouette Test for Adolescents (TSA; Cruz and Maganto, 2003). This Spanish instrument, adapted for the adolescent population, consists of eight male and eight female figures with progressively increasing body sizes. The patient was requested to select the figure that she perceived to reflect her own body size and the one that she desired. Then, according to her BMI, the real silhouette was also selected. BD (TSA-I) was assessed by calculating the discrepancy between the perceived and desired body sizes. Body image distortion (TSA-D) was assessed by calculating the discrepancy between the perceived and real body sizes. The possible range of scores was between **−**3 and 3. This questionnaire presents good validity and good reliability indices.

#### Body-Related Attentional Bias

- Attentional bias was measured as the total fixation time (evaluated in milliseconds) and the total number of fixations on weight-related and non-weight-related body parts. In accordance with the PASTAS, weight-related areas of interest (AOIs) were defined as the thighs, buttocks, hips, stomach, legs and waist. The remaining body parts (i.e., head, shoulders, arms, décolletage, neck and chest) were labeled as non-weight-related AOIs.

#### Visual Analog Scales (VAS)

- Full-body illusion (FBI) was assessed at the beginning of each session using a visual analog scale (VAS) to estimate the intensity of the illusion from 0 to 100.
- Fear of gaining weight (FGW) and body-related anxiety were assessed on a VAS from 0 to 100 at the beginning and end of each exposure therapy session.
- Anxiety related to specific body parts (to which the patient was exposed) was assessed every 30 s throughout each session.

### Instruments

The patient was exposed to an immersive virtual scenario through a VR head-mounted display (HMD-HTC-VIVE). In addition to the two controllers that HTC-VIVE usually provides, three body trackers were also used to track full-body motion. Furthermore, VR HMD FOVE Eye Tracking was used to detect and register eye movements. The headset uses incorporated position and orientation eye-tracking systems.

Virtual avatars were created with Unity 3D and Blender 2.78, integrating all the components within the virtual environment. The virtual environment was a simple room with a large mirror on the front wall, and without any furnishings. The mirror was large enough to reflect every limb of the body and was placed 1.5 m in front of the patient. A young female avatar wearing a basic white t-shirt, blue jeans and black trainers was created. The avatar also wore a swimming cap to avoid any effects of hairstyle.

### Procedure

#### Overview

Prior to treatment, written informed consent was obtained from both the patient and her parents. The VR body exposure intervention consisted of five sessions. Before the first session, there was a pre-assessment session on March 25th, 2019. One week after the last session, the post-assessment was performed on May 6th, 2019. This post-assessment was repeated approximately 5 months after the end of the treatment on September 25th, 2019. The treatment was administered by two general health psychologists with clinical experience in the treatment of adolescents.

#### Pre-assessment

In the pre-assessment session, which lasted approximately 1 h, the virtual avatar was generated by taking a frontal and lateral photograph of the patient and creating an avatar whose silhouette matched the pictures by adjusting the different parts of the silhouette according to the photographs. In the meantime, the other therapist administered the pre-assessment questionnaires and answered the patient’s questions.

**FIGURE 2:**
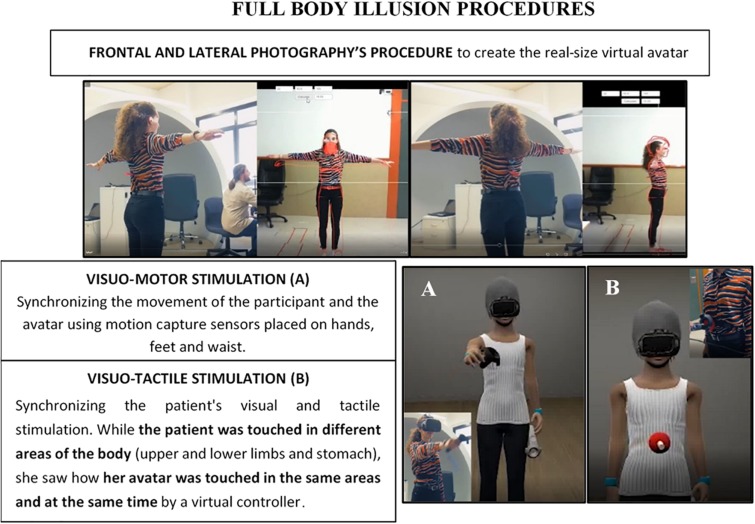
Description and illustration of the procedures conducted to create the avatar and induce a full-body illusion (FBI).

Next, the FBI was induced by using two different procedures, visuo-motor and visuo-tactile stimulation (see Figure 2 and Supplementary Video S1 for more details). Both procedures lasted 3 min. Once inside the virtual environment, the patient was able to observe herself in the first-person perspective and look at herself in a mirror (in the third-person perspective).

Once the FBI was induced, the intensity of the FBI, body-related anxiety and the FGW were assessed with the VASs. To assess body-related attentional bias, calibration and recording procedures were conducted using VR HMD FOVE Eye Tracking. The patient’s gaze was tracked while she was asked to observe her virtual body in the mirror for 30 s [a similar recording time to that used in the studies of Jansen et al. (2005) and Roefs et al. (2008)]. During this process and as a cover story, the patient was told to remain still while the virtual avatar position was being recalibrated.

#### Body Exposure Therapy Sessions

Each session, which lasted approximately 1 h and took place once a week, began by inducing a body ownership illusion using the procedures mentioned above and the VASs. The exposure treatment was initiated using a virtual body with the same BMI as the patient. During the following sessions, the BMI of the avatar was progressively increased (by a BMI score of 0.20 kg/m^2^ per session) until the target weight (healthy BMI) was reached.

During exposure to each avatar, the patient was asked to focus on different parts of the virtual body. To facilitate this task, the part of the body to which the participant was being exposed was illuminated. The order of exposure to the different parts of the body was established from the scores obtained in the PASTAS, starting with the body parts that produced the least amount of anxiety before moving on to those producing more anxiety. The level of anxiety experienced was evaluated every 30 s using a VAS. When the level of anxiety had fallen by 40% with respect to the initial measurement, treatment continued, with the patient being exposed to the next body part.

Once the patient had been exposed to all parts of the body, she was asked about her anxiety regarding her whole body. When her anxiety levels had decreased by 40% with respect to the initial measurement, the session was terminated. At the end of each session, the patient was exposed to a relaxing VR environment (forest or garden) for 5 min.

Each of the following treatment sessions began with an avatar with a progressively increasing BMI according to the hierarchy. In the cases where whole-body anxiety had not fallen by 40% or if the exposure to various parts of the body had not been completed, the same avatar as that of the previous session was used.

### Data Analysis

The OGAMA (Open Gaze and Mouse Analyzer) software was used to transform the raw eye-tracking data into suitable quantitative data. An additional data transformation was conducted by subtracting the difference between weight-related and non-weight-related AOIs (e.g., for the number of fixations, 25 W-AOIs–10 NW-AOIs = 15). Therefore, a positive outcome would mean that the participant had been looking more at the weight-related body parts than at the non-weight-related body parts, while a negative outcome would mean the opposite.

In addition, the results of the self-report scales from the pre-, post- and follow-up assessments and the data recorded from each session with the different VASs are described in detail in the Results section. Reliable changes and clinically significant changes were calculated for the post-assessment and follow-up assessment measurements, following the guidelines of Jacobson and Truax (1991) and using the Leeds Reliable Change indicator calculator in Excel (Morley and Dowzer, 2014) for single cases. Reliable changes and clinically significant changes were calculated only for the measures with clinical and/or community norms available (e.g., BMI, EDI-DT, EDI-BD, and PASTAS). Accordingly, analyses were conducted by using clinical and community means and standard deviations, provided by the original sources for each measure (Reed et al., 1991; Elosua et al., 2010).

These analyses were not conducted for the measures with no available clinical and/or normative norms (e.g., TSA, VASs, and body-related attentional bias).

## Results

### VR Body Exposure Therapy Sessions

**FIGURE 3:**
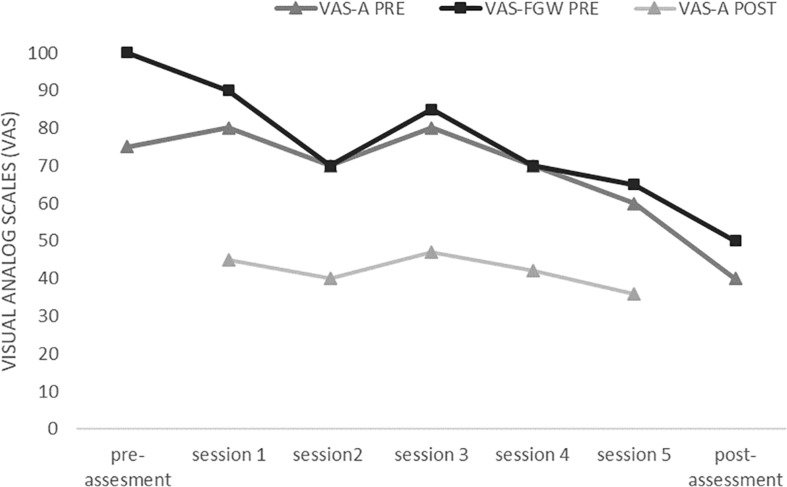
Initial values for fear of gaining weight (VAS-FGW pre) and body anxiety (VAS-A pre) and the final value for body anxiety (VAS-A post) after the five VR sessions.

As shown in Figure 3, both VAS-A and VAS-FGW scores gradually decreased over the five exposure sessions and from the pre- to post-assessment sessions. According to these results, the patient completed the hierarchy of the exposure, indicating that the level of anxiety experienced regarding each body part and the whole body had decreased by at least 40% in each session. At the post-assessment, the levels of body anxiety and the FGW were considerably lower than those of the pre-assessment.

**FIGURE 4:**
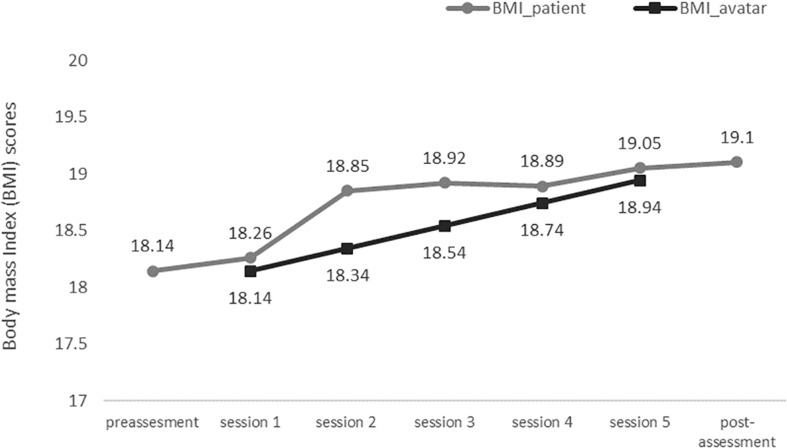
Body mass index (BMI) values of the avatar in the five VR sessions, as well as those of the patient in the pre- and post-treatment assessments.

According to the VAS assessing the FBI (VAS-FBI), the values did not vary greatly across the five exposure sessions, ranging from 80 to 85. These values indicated high FBI rates. Finally, as can be observed in Figure 4, there was a gradual increase in the patient’s BMI, following a similar pattern to the pre-established gradual increase of the BMI of the avatar. Both the patient and the avatar reached the target healthy BMI of 19 (BMI percentile: 25th) that had been established by the clinicians treating the patient.

### Pre-assessment, Post-assessment and 5-Month Follow-Up

As shown in Table 1, there was a significant reduction in all the measurements between the pre- and post-assessment sessions, specifically in the drive for thinness (EDI-DT) and BD (EDI-BD), body anxiety (PASTAS), body image disturbances (TSA-Distortion and TSA-Dissatisfaction), and the FGW and body anxiety (VAS-FGW and VAS-A). Moreover, at the end of the intervention, there were reliable improvements in the EDI-DT, EDI-BD, and PASTAS scores and BMI level, as well as clinically significant improvements in the PASTAS score and BMI level (see Table 1).

**TABLE 1:** Results from the pre-treatment, post-treatment and 5-month follow-up assessments.

Measures	Pre-treatment session	Post-treatment session	5-month follow-up
BMI	18.14	19.10^a, b^	19.36
EDI-DT	20	14	16
EDI-BD	29	18^a^	14
PASTAS	21	15^a,b^	13
TSA-Distortion	*3*	*2*	*1*
TSA-Dissatisfaction	*0*	**−***1*	**−***1*
VAS-A	*75*	*40*	*40*
VAS-FGW	100	50	80
Complete fixation time (ms)	5150	**−**5059	**−**2068
Number of fixations	23	**−**11	2

*Body Mass Index (BMI), Eating Disorder Inventory (EDI-3), Drive for Thinness (DT), Body Dissatisfaction (BD), Physical Appearance State and Trait Anxiety Scale (PASTAS), Silhouette Test for Adolescents (TSA), Visual Analog Scale (VAS) of Body Anxiety (A), Fear of Gaining Weight (FGW) and Body-Related Attentional Bias. Reliable changes and clinically significant changes were only calculated for the measures with clinical and/or community norms available (e.g., BMI, EDI-DT, EDI-BD, and PASTAS). ^a^Reliable change.^b^Clinically significant change.*

After 5 months, similar outcomes were obtained to those of the post-treatment assessment, with a slight increase in the BMI and reductions in body dissatisfaction, body image distortion and body anxiety levels. Drive for thinness and especially the FGW were increased in the follow-up assessment compared to the post-assessment. However, the slight improvements or deteriorations observed among some of the symptoms (EDI, PASTAS or BMI) between the post-assessment and follow-up assessment were neither reliable nor clinically significant (see Table 1). Furthermore, the BMI of the patient was maintained after one year, with a BMI of 18.9 kg/m^2^ recorded on February 18th, 2020.

Regarding attentional bias, as can be observed in Table 1, scores were positive for both measures in the pre-assessment, indicating that the patient showed an attentional preference for weight-related body parts. After the intervention, there was a significant decrease in both measures, indicating an attentional preference for non-weight-related AOIs. This change in attentional bias was especially noticeable for the complete fixation time, with the patient focusing her attention for longer on the non-weight-related AOIs.

After 5 months, a similar gaze pattern was observed, with a longer amount of time spent on non-weight-related AOIs and an equal number of fixations on weight-related and non-weight-related AOIs.

## Discussion

This study provides preliminary evidence of the usefulness of a VR software in improving the effects of classic CBT in AN. This VR body exposure therapy first focused on reducing the FGW and associated body anxiety. As mentioned in the Results section, the FGW decreased progressively over the five VR sessions, resulting in a reduction of 50% by the end of the intervention. The patient successfully completed all the steps in the hierarchy within each session (e.g., showing reduced anxiety toward weight-related body parts by at least 40%). Consequently, whole body-related anxiety also fell substantially.

Our results are in line with those of a previous study (Levinson et al., 2014) that reported a decrease in AN symptomatology after five sessions of imaginal exposure. However, the current study overcame some of the previously reported limitations of imaginal exposure. Firstly, VR technology allowed the creation of a virtual body with the same silhouette and BMI as those of the participant, generating a full-body illusory experience of owning this virtual body. Furthermore, controlled BMI increases of the avatar were performed between subsequent sessions, leading to a specifically targeted healthy BMI that had been agreed upon previously by the clinicians treating the patient.

At the end of the body exposure intervention, reductions were observed not just in the FGW and related body anxiety levels, but also in other important AN symptoms such as BD, drive for thinness and body image distortion. In addition, the low BMI, another important diagnostic criterion for AN (American Psychiatric Association [APA], 2013), had increased by the end of the treatment, surpassing the level of 18.5 defined as “healthy” by the World Health Organization (World Health Organization [WHO], 2004). Our results for BD were also in line with those of previous studies using mirror-exposure techniques to reduce BD in ED patients (Key et al., 2002; Hildebrandt et al., 2012) and in women with high levels of BD (Jansen et al., 2016). Modifying disturbed body representations, including the perceptual and affective components, is an important target in the treatment of AN. The brain creates multisensory representations of the body and the environment where it is placed (body matrix; Moseley et al., 2012). New technologies might help to improve disturbances in stored implicit and explicit body representations by allowing the brain to correct mismatches (e.g., in perceptual information regarding the body) and update dysfunctional content of the body matrix in a paradigm known as embodied medicine (Riva, 2018). The use of VR full-body illusion techniques (e.g., by changing the size of the virtual body) has already shown promising results in changing dysfunctional body representations in individuals with AN (Keizer et al., 2016; Serino et al., 2019). Our results provide further support for this innovative approach.

As for attentional bias, the patient showed an attentional preference for weight-related body parts in the pre-assessment, which is in line with previous studies conducted on adolescents with restrictive AN (Bauer et al., 2017). However, at the end of the intervention and in the follow-up session, the patient showed greater focus on non-weight-related body areas. These results are in accordance with a previous study (Jansen et al., 2016) that used a similar exposure procedure (unattractive body parts) over five sessions and observed higher levels of body satisfaction and reduced body checking behaviors. However, while Jansen et al. (2016) assessed body checking and avoidance behaviors via a questionnaire, we used an eye-tracking device in this study to obtain a continuous and more objective measure of attentional bias.

Finally, at the 5-month follow-up assessment, almost all the changes observed in the post-assessment were maintained, with a slight increase in BMI values and slight reductions in body dissatisfaction, body image distortion and body anxiety levels. The patient also showed similar gaze patterns as in the post-assessment, with a preference for attending non-weight-related body parts. Surprisingly, the FGW increased in the follow-up assessment compared to the post-assessment, but it did not reach the same high levels as those observed in the pre-assessment. The small increase in BMI values observed 5 months after the treatment ended may have elicited an increase in the FGW and even a small increase in the drive for thinness. However, other body-related measures, such as body image disturbances, body anxiety and body-related attentional bias, were not affected.

This study had some limitations that should be mentioned. First, it was based on a single patient who received VR treatment as an add-on to treatment as usual (CBT). Therefore, we cannot conclude that the success of the intervention was due to the VR exposure *per se*. Other limitations regarding the VR procedure included the discrepancy between the current BMI of the patient and the BMI of the avatar. As the increase in the patient’s BMI was steeper than that calculated for the avatar, the patient was exposed to an avatar that had a lower BMI than the patient herself, especially in sessions 2 and 3. In addition, even though the FGW levels were notably reduced after treatment, more VR sessions might have been needed to obtain a long-term reduction of this core fear in AN. These additional VR sessions may be particularly useful for those patients who recover a healthy BMI in order to prevent future relapses, which are commonly observed among individuals with AN (Zipfel et al., 2015). Finally, a longer follow-up assessment, for instance, one year after the intervention, would have been more useful to better understand the development of the symptomatology in the patient.

Regarding the patient’s perspective, she was quite motivated to get involved in the therapy since it was the first time that she had been exposed to VR. After the intervention, she described the sessions as being not only entertaining, but also useful in helping her confront her FGW and body anxiety. Furthermore, the patient noted that she felt progressively more relaxed during the session and noticed the illuminated body part less, thus helping her to focus her attention on other parts of the avatar (e.g., non-weight-related body parts). However, the patient reported that the virtual room and the clothes of the avatar were too bland, the avatar did not look realistic enough, and she would have preferred the avatar not to be wearing a swimming cap.

To confirm the efficacy of the VR body exposure therapy, controlled clinical trials should be conducted. Our group has already started one such study (clinicaltrials.gov, NCT 04028635), a randomized controlled clinical trial in which we have increased the sample size and are comparing CBT with VR exposure to CBT alone. The present study showed that supplementing CBT with a VR-based exposure to a virtual body with a progressively increasing BMI was well accepted by a patient with restrictive AN, who not only reduced her ED symptomatology, but also reached and maintained a healthy BMI.

## Data Availability Statement

The datasets generated for this study are available on request to the corresponding author.

## Ethics Statement

The studies involving human participants were reviewed and approved by Ethical Committee of the University of Barcelona. Written informed consent to participate in this study was provided by the participant and by the participant’s legal guardian/next of kin. The patient and the patient’s legal guardian gave permission to publish the material anonymously.

## Author Contributions

BP-G, ES-T, MF-G, and JG-M designed and conceptualized the study. BP-G and NF-P conducted the research, participated in the clinical sessions, and conducted the formal analysis. ES-T, MC-R, PS-U, MF-G, and JG-M supervised the whole process and provided the necessary resources during the clinical sessions. BP-G, LY, and YO prepared the original draft. NS, ES-T, MC-R, PS-U, MF-G, and JG-M contributed to the critical review and editing of the manuscript. BP-G and NS corrected the revisions of the final version of the manuscript. JG-M was responsible for the project coordination and the funding.

## Conflict of Interest

The authors declare that the research was conducted in the absence of any commercial or financial relationships that could be construed as a potential conflict of interest.

## Supplementary Material

VIDEO S1 Brief description of the procedures conducted to create the avatar and induce a full-body illusion (FBI) during the pre-assessment and five-exposure sessions.
